# Advances in Corrosion Mechanisms, Surface Engineering, and Protective Technologies for Metallic Materials: Recent Progress and Future Perspectives

**DOI:** 10.3390/ma19204401

**Published:** 2026-10-20

**Authors:** Luca Pezzato

**Affiliations:** Institute of Condensed Matter Chemistry and Energy Technologies, National Research Council of Italy, 35127 Padova, Italy; luca.pezzato@cnr.it

## 1. Introduction

Metallic materials are the most important materials in engineering applications. However, their performance is often limited by corrosion and environmentally induced degradation. The economic impact of corrosion affects virtually every industrial sector, including transportation, marine engineering, energy generation, biomedical devices, and aerospace applications [1].

The papers included in this collection, selected as Editor’s Choice Articles in the “Corrosion” Section of *Materials*, illustrate the multidisciplinary character of contemporary corrosion science. They cover fundamental aspects of degradation mechanisms, advanced surface engineering technologies, innovative coating solutions, emerging alloy concepts, and strategies for enhancement under increasingly severe operating conditions [Contributions 1–13]. The studies span a broad range of materials and systems, including stainless steels, titanium alloys, nickel-based superalloys, magnesium alloys, high-entropy alloys, aluminum alloys, reinforced cementitious composites, and additive-manufactured components. Particular attention is devoted to environmentally assisted degradation phenomena, such as stress corrosion cracking, hydrogen embrittlement, atmospheric corrosion, and ammonia-induced corrosion, as well as to the development of functional coatings and bio-inspired protective layers. Emerging approaches involving layered double hydroxides, ZnO thin films, laser-cladded high-entropy coatings, peening technologies, and microstructure-tailored alloys demonstrate the growing trend toward multifunctional and intelligent corrosion protection systems. Collectively, these contributions emphasize the need for an integrated understanding of corrosion processes across multiple length scales, from atomistic mechanisms to service-scale performance. The findings presented in this collection provide valuable insights into future developments in durable, sustainable, and high-performance metallic systems operating in increasingly demanding environments.

## 2. Understanding Corrosion Mechanisms Across Multiple Scales

A major theme emerging from this collection is the increasingly sophisticated understanding of corrosion phenomena at multiple length scales [2].

Felix et al. investigated stress corrosion cracking through atomistic simulations and demonstrated the critical role of water-assisted dissolution mechanisms in oxide materials. Their findings provide new insights into crack-tip evolution and environmentally induced damage in protective oxide films [3].

Complementary experimental work by Li et al. examined atmospheric corrosion through long-term monitoring of rust-layer evolution and critical humidity thresholds. The study demonstrated that both rust composition and morphology strongly affect corrosion initiation and propagation in outdoor environments [Contribution 6].

Environmentally assisted degradation was also investigated under emerging service conditions. Jose et al. comprehensively reviewed chloride-induced stress corrosion cracking (CISCC) in nuclear dry storage canisters and highlighted advanced peening-based mitigation strategies [Contribution 2]. Likewise, Damm et al. reported novel observations concerning hydrogen-dislocation interactions at cryogenic temperatures, revealing suppressed hydrogen embrittlement despite persistent hydrogen effects on plastic deformation mechanisms [Contribution 12].

The growing importance of alternative energy systems is reflected in the work of Liu et al., who explored the corrosion behavior of Ni80A alloy valves operating in ammonia-rich environments relevant to future ammonia-fueled marine engines [Contribution 8].

## 3. Surface Engineering as a Key Corrosion Mitigation Strategy

Surface engineering continues to represent one of the most effective routes for extending component lifetime and improving corrosion resistance [4,5].

Adomniței et al. demonstrated the beneficial effects of atmospheric plasma-sprayed yttria-stabilized zirconia coatings deposited on Inconel 718. The combined ceramic and metallic bond-coat architecture improved electrochemical corrosion resistance and provided additional protection against aggressive environments [Contribution 1].

Peening-based technologies were extensively discussed by Jose et al., who emphasized the ability of shot peening, ultrasonic impact peening, and laser shock peening to introduce compressive residual stresses capable of mitigating stress corrosion cracking in critical nuclear storage systems [Contribution 2].

Similarly, Walczak et al. investigated shot-peened Ti6Al4V alloys produced through conventional and additive manufacturing routes. Their results highlighted the complex relationship between microstructure, surface roughness, residual stresses, and corrosion behavior [Contribution 5].

The significance of surface finishing for additively manufactured components was further demonstrated by Ahuir-Torres et al. Centrifugal mass finishing substantially reduced surface roughness and improved corrosion resistance in additively manufactured 17-4PH stainless steel exposed to chloride-containing environments [Contribution 13].

## 4. Emerging Protective Coatings and Functional Surface Layers

One of the strongest trends represented in the collection is the development of advanced protective coatings capable of providing multifunctional performance [6,7].

Cao et al. reviewed layered double hydroxide (LDH)-based coatings and highlighted their unique ability to combine barrier protection, ion exchange, chloride trapping, hydrophobicity, and self-healing capabilities [Contribution 4]. These features position LDHs among the most promising candidates for next-generation smart corrosion protection systems.

ZnO thin films deposited by atomic layer deposition also demonstrated remarkable protection potential. Kania et al. showed that increasing ZnO thickness significantly enhanced corrosion resistance in biodegradable magnesium alloys intended for biomedical applications [8].

High-entropy alloy coatings represent another rapidly developing research area. Ao et al. demonstrated that the boron microalloying of laser-cladded AlCoCrFeNiMoTi coatings promoted in situ TiB_2_ formation, improving hardness, wear resistance, and corrosion behavior when optimized compositions were employed [Contribution 11].

In addition, Jin et al. investigated ultrasonic-assisted stripping of aluminum coatings from cobalt-based superalloys. The application of ultrasonic energy accelerated coating removal while maintaining substrate integrity, opening new opportunities for the maintenance and refurbishment of high-value aerospace components [Contribution 10].

## 5. Alloy Design and Microstructural Optimization

Beyond coatings and surface modifications, alloy development remains a cornerstone of corrosion engineering [9].

Lee et al. demonstrated that the incorporation of Cr_2_N and optimization of Ni/Mn ratios in HIP-sintered 316L stainless steel can simultaneously improve corrosion resistance and mechanical properties. Their work illustrates how microstructural engineering can reduce reliance on expensive alloying elements while maintaining high performance [Contribution 7].

New alloy concepts were also explored through the development of magnesium-containing multi-principal element alloys. Helmer et al. reported enhanced passivation behavior associated with TiO_2_-rich surface films, resulting in promising corrosion resistance and mechanical performance [Contribution 9].

The increasing interest in compositional complexity is further evidenced by the success of high-entropy alloy systems, where tailored phase formation and microstructural refinement contributed to superior surface durability [Contribution 11].

Beyond metallic alloys and coatings, corrosion durability remains critical in structural materials containing embedded steel reinforcements. The study on rebar-reinforced ultra-high-performance concrete (UHPC) investigated the influence of corrosion on both degradation processes and the subsequent tensile behavior of reinforced systems [10]. The results emphasized the necessity of considering not only corrosion resistance but also the residual mechanical integrity of engineering structures after exposure to aggressive environments. Such investigations are particularly relevant for infrastructure applications, where long-term structural reliability depends on the coupled interaction between corrosion damage and mechanical performance [10].

## 6. Bio-Inspired and Sustainable Approaches

The transition toward sustainable corrosion protection strategies is another important theme emerging from this collection [11].

Jaume et al. demonstrated that naturally occurring marine microorganisms can promote the formation of protective surface layers on 5083 aluminum alloy, providing significant resistance against pitting corrosion [Contribution 3]. Such findings support the development of environmentally friendly alternatives to conventional chromate-based protection technologies.

The sustainable design of corrosion-resistant materials is also reflected in the development of self-healing coatings, ion-exchange systems, and multifunctional protective architectures designed to reduce maintenance requirements and extend service lifetime [Contribution 4].

## 7. Emerging Trends and Future Directions

Several key trends can be identified from the contributions collected in this issue:

1. The increasing integration of atomistic modeling and experimental validation to understand corrosion mechanisms [3] [Contribution 6].
2. Increasing attention toward performance-based durability assessment, where corrosion damage is directly correlated with residual mechanical properties and structural integrity in service environments [10].
3. The development of multifunctional smart coatings combining barrier, self-healing, and active protection mechanisms [Contribution 4] [8].
4. The expansion of additive manufacturing technologies and associated corrosion challenges [Contributions 5, 13].
5. A growing focus on materials for hydrogen and ammonia-based energy systems [Contributions 8, 12].
6. The increased utilization of advanced surface engineering techniques such as peening, laser cladding, and atomic layer deposition [Contributions 1, 2, 11] [8].
7. The development of environmentally sustainable and bio-inspired protection approaches [Contributions 3, 4].
8. The continued exploration of compositionally complex alloys, including high-entropy and multi-principal element systems [Contributions 9, 11].

Although significant advances have been made in corrosion protection technologies, several challenges remain. Future research should focus on improving the long-term stability and self-healing ability of protective coatings under complex service environments, enhancing the predictive accuracy of corrosion models through artificial intelligence and data-driven approaches, and developing standardized accelerated testing methods that more closely replicate real-world conditions. In addition, further efforts are needed to understand the interactions between alloying elements, corrosion products, and environmental factors at multiple length scales, as well as to develop cost-effective and environmentally friendly protection systems that comply with increasingly stringent sustainability requirements. The integration of advanced manufacturing, digital materials design, real-time monitoring technologies, and intelligent corrosion management strategies is expected to play a key role in overcoming these challenges and improving the durability and reliability of future engineering systems.

## 8. Conclusions

The papers selected as the Editor’s Choice in the Corrosion section of *Materials,* and collected in this editorial, provide an excellent overview of the current advances in corrosion science, surface engineering, durability assessment, and protective technologies for metallic materials and engineering systems [Contributions 1–13].

From atomistic studies of oxide dissolution and stress corrosion cracking [3] to advanced coatings [Contributions 1, 4, 11] [8], optimized alloy design [Contributions 7, 9], additive-manufactured materials [Contributions 5, 13], corrosion effects in reinforced UHPC structures [10], bio-inspired protection concepts [Contribution 3], and materials for future hydrogen and ammonia infrastructures [Contributions 8, 12], these contributions illustrate both the breadth and depth of modern corrosion research.

Collectively, these studies demonstrate that future corrosion mitigation strategies will increasingly rely on integrated approaches combining materials design, surface engineering, advanced manufacturing, predictive modeling, and sustainability principles. Such developments are expected to contribute significantly to the reliability, safety, and longevity of metallic systems operating in the demanding technological environments of the future.
